# Propolis Flavonoids Ameliorate Chronic Osteomyelitis in a Rat Model by Modulating TAZ‐Regulated Treg/Th17 Balance

**DOI:** 10.1002/iid3.70432

**Published:** 2026-06-04

**Authors:** Yujie Wang, Chaozhu Zhang, Zeyang He, Liqiang Li

**Affiliations:** ^1^ Fourth Department of Orthopedics and Traumatology Hebei Hospital of Traditional Chinese Medicine Shijiazhuang China; ^2^ Department of Orthopedics Hebei Hospital of Traditional Chinese Medicine Shijiazhuang China

**Keywords:** chronic osteomyelitis, immunomodulation, Propolis flavonoids, TAZ, Treg/Th17 balance

## Abstract

**Objective:**

To investigate the therapeutic mechanism of propolis flavonoids (PF) in chronic osteomyelitis (COM) through transcriptional coactivator with PDZ‐binding motif (TAZ)‐mediated regulation of regulatory T cell (Treg)/T helper 17 (Th17) cell homeostasis.

**Methods:**

Sixty 3‐month‐old Wistar rats were randomly divided into control, model, and PF‐treated groups. In vitro, human peripheral blood mononuclear cells (PBMCs) and rat PBMCs were stratified into five treatment groups: control (10% healthy control (HC) serum), osteomyelitis model (15 ng/mL transforming growth factor‐β1 TGF−β1 + 10% HC serum), model+PF 15ng/mLTGF−β1+10µg/mLPF, TEA domain transcription factor 1 (TEAD1)+model TGF−β1+25nMTEAD1, and TEAD1+model+PF. TAZ signaling components (TAZ, RAR‐related orphan receptor gamma t (RORγt), Forkhead box P3 (FOXP3)), inflammatory cytokines (interleukin (IL)−17, IL‐21, IL‐2, IL‐10), and T cell subsets (Th17/Treg ratios) were analyzed via Western blot, enzyme‐linked immunosorbent assay (ELISA), and flow cytometry. Periosteal cell proliferation was quantified using 5‐ethynyl‐2’‐deoxyuridine (EdU) assay. Bacterial load in bone tissue was quantified using colony‐forming unit (CFU) counts.

**Results:**

PF administration significantly upregulated TAZ protein expression compared to untreated osteomyelitis models (*p* < 0.05). PF treatment significantly reduced bacterial burden in bone tissue (*p* < 0.001). In the PF group, Th17 cell proportions increased (2.28 ± 0.35%) compared to the osteomyelitis model group (1.58 ± 0.31%) and RORγt expression was elevated, coupled with reduced Treg populations (1.66 ± 0.27%) compared to the osteomyelitis model group (11.5 ± 0.29%) and reduced FOXP3 levels. Notably, pro‐inflammatory cytokines IL‐17 and IL‐21 decreased in PF‐treated groups, while anti‐inflammatory IL‐10 and IL‐2 increased. Periosteal cell proliferation rates improved significantly with PF intervention. TEAD1 overexpression attenuated PF‐mediated effects in both human and rat PBMCs, confirming TAZ's central regulatory role across species.

**Conclusion:**

Propolis flavonoids ameliorate chronic osteomyelitis through TAZ‐dependent mechanisms that rebalance Treg/Th17 polarization and promote bacterial clearance. This involves restoring Th17 cell populations while concurrently suppressing their key effector cytokines, ultimately reducing inflammatory pathology and promoting bone repair.

## Graphical abstract

This diagram illustrates the mechanism by which Propolis Flavonoids (PF) ameliorate Chronic Osteomyelitis (COM). PF upregulates the transcriptional coactivator TAZ, which is suppressed in COM. Elevated TAZ restores Treg/Th17 homeostasis by promoting the Th17‐master regulator RORγt while suppressing the Treg‐specific transcription factor FOXP3. This cellular rebalancing decreases pro‐inflammatory cytokines (IL‐17, IL‐21) and increases anti‐inflammatory cytokines (IL‐10, IL‐2), ultimately reducing inflammation and promoting bone repair. This therapeutic effect is antagonized by the TAZ inhibitor, TEAD1.

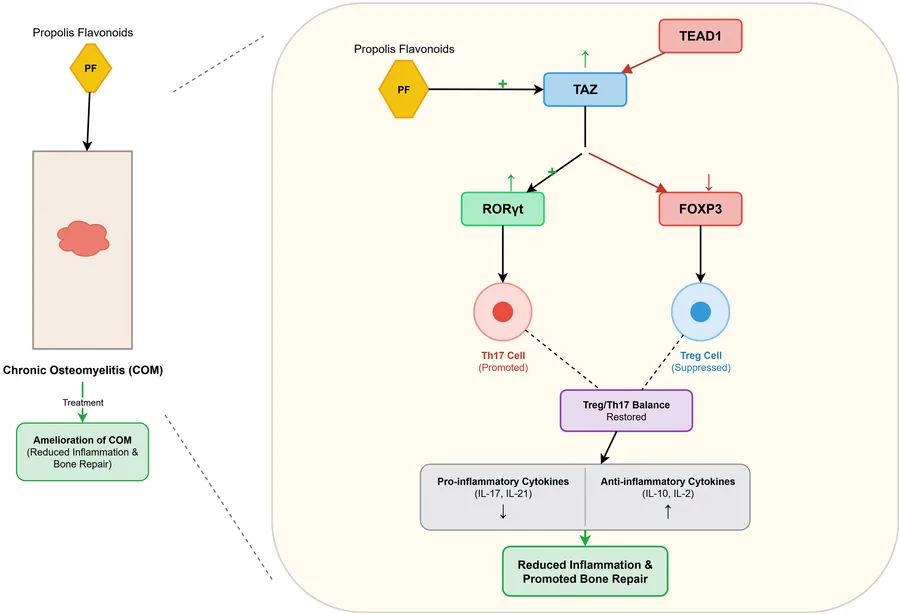

**Abbreviations**
ANOVA: analysis of variance
COM: chronic osteomyelitis
EdU: 5‐ethynyl‐2′‐deoxyuridine
ELISA: enzyme‐linked immunosorbent assay
FOXP3: Forkhead box P3
IL: interleukin
PBMCs: peripheral blood mononuclear cells
PF: propolis flavonoids
RORγt: RAR‐related orphan receptor gamma t
SD: standard deviation
TAZ: transcriptional coactivator with PDZ‐binding motif
TEAD1: TEA domain transcription factor 1
TGF‐β1: transforming growth factor‐β1
Th17: T helper 17 cell
Treg: regulatory T cell

## 1 Introduction

Chronic osteomyelitis (COM), characterized by persistent bone infection and inflammation, poses significant clinical challenges due to its complex pathophysiology and recalcitrant nature [1, 2, 3]. This highlights the urgent need for multifaceted therapies that not only control infection but also promote tissue regeneration, a field where advanced biomaterials like functionalized hydrogels and scaffolds are showing significant promise [4, 5]. Predominantly triggered by microbial invasion (commonly *Staphylococcus aureus*), this condition necessitates prolonged antibiotic regimens and repeated surgical debridement, with treatment failure rates exceeding 20% in refractory cases [6]. Beyond its substantial socioeconomic burden, COM severely compromises patients' quality of life through chronic pain, pathological fractures, and systemic complications [7]. Emerging evidence underscores the pivotal role of immune dysregulation in COM progression. Bacterial pathogens, particularly *S. aureus*, can actively manipulate the host immune system to establish persistence, often by disrupting the critical balance between pro‐inflammatory T helper 17 (Th17) cells and immunosuppressive regulatory T cells (Tregs) [8, 9]. Recent high‐impact research highlights that immune cells in chronic inflammatory environments, such as rheumatoid arthritis, exhibit significant plasticity regulated by local signals, suggesting that targeting these regulatory mechanisms can attenuate inflammation without compromising host defense [10].

The Th17/Treg axis serves as a critical immunological rheostat in bone homeostasis. Th17 cells, driven by RAR‐related orphan receptor gamma t (RORγt)‐mediated differentiation, exacerbate inflammatory bone destruction through interleukin (IL)−17/IL‐21 secretion, while Forkhead box P3 (FOXP3)+ Tregs counterbalance this process via IL‐10/IL‐2 production to maintain immune tolerance [11, 12, 13]. Clinical studies reveal that COM patients exhibit skewed Th17/Treg ratios correlating with disease severity, suggesting therapeutic potential in modulating this equilibrium [8, 14]. However, conventional immunomodulators often lack specificity in targeting this axis without systemic immunosuppression.

Propolis flavonoids (PF) are a complex mixture of bioactive polyphenolic compounds derived from propolis, a resinous substance collected by bees from various plant sources [15]. Propolis and its flavonoid components have garnered considerable attention for their wide spectrum of biological activities, including well‐documented anti‐inflammatory, antioxidant, antimicrobial, and immunomodulatory properties [16, 17]. Alongside host‐directed immunotherapies, novel strategies targeting bacterial biofilms, such as targeted nanoparticle‐based sonodynamic therapy, are emerging as promising complementary approaches [18]. Mechanistic studies indicate their capacity to suppress NF‐κB signaling in macrophages and enhance osteoblast mineralization [17, 19]. These properties make PF a promising candidate for addressing inflammatory conditions like COM. Notably, preliminary data suggest PF may regulate T cell polarization through Hippo pathway components, particularly the transcriptional coactivator with PDZ‐binding motif (TAZ) ‐ a molecular switch governing cellular differentiation and immune responses [20]. The Hippo‐TAZ signaling pathway is increasingly recognized for its role in immunity and inflammation, influencing T cell differentiation and function [21]. TAZ modulates nuclear translocation of TEA domain transcription factor (TEAD) transcription factors to regulate genes critical for Th17 (RORγt) and Treg (FOXP3) differentiation [22, 23]. Specifically, TAZ has been shown to promote Th17 differentiation [24], while the Treg/Th17 balance is crucial in COM, as indicated by studies such as Ge et al. (2023) linking TAZ‐mediated regulation of this balance to COM pathogenesis [8]. Despite these insights, the precise interplay between PF, TAZ signaling, and Th17/Treg dynamics in COM remains incompletely explored. The primary novelty of this study lies in elucidating a specific molecular link between PF, the Hippo‐TAZ pathway, and a nuanced regulation of the Treg/Th17 axis in COM, moving beyond general anti‐inflammatory descriptions.

While previous studies, such as Ge et al. (2023), established TAZ as a regulator of the Treg/Th17 axis in COM [8], the specific pharmacological modulation of this pathway by natural compounds remains unexplored. The primary novelty of this study lies in elucidating PF as a specific modulator of the Hippo‐TAZ pathway and, more importantly, revealing a phenomenon of functional plasticity where PF uncouples Th17 cell proliferation from pathogenic cytokine production. This study aims to bridge this knowledge gap by systematically investigating PF's immunomodulatory mechanisms in COM pathogenesis. We hypothesize that PF attenuates chronic osteomyelitis through TAZ‐mediated restoration of Th17/Treg homeostasis. By integrating an in vivo rat model of COM to observe systemic effects and in vitro models using both human and rat PBMCs to dissect specific molecular pathways, we seek to provide a comprehensive mechanistic rationale for PF's therapeutic potential in COM. These findings are anticipated to provide a mechanistic foundation for developing PF‐based therapies targeting immune‐bone crosstalk in COM management.

## 2 Materials and Methods

### 2.1 Experimental Materials

#### 2.1.1 Experimental Animals and Cells

Sixty healthy male Wistar rats (3‐month‐old, 300‐350 g) were obtained from the Animal Research Center and housed in a controlled environment (22 ± 2°C, 45 ± 5% humidity, 12 h light/dark cycle) with free access to standard chow and water. Randomization of animals into groups was performed using a simple random sampling method (random number generator). Prior to experimental procedures, animals were anesthetized via intraperitoneal injection of pentobarbital sodium (50 mg/kg) with depth of anesthesia confirmed by absence of pedal reflex. Euthanasia was performed by cervical dislocation in accordance with AVMA Guidelines (2020 edition) following confirmation of deep anesthesia. All experimental protocols were approved by the Institutional Animal Care and Use Committee of Hebei Hospital of Traditional Chinese Medicine and conducted in compliance with NIH Guide for the Care and Use of Laboratory Animals (8th ed.) and ARRIVE Guidelines 2.0 [25]. The experimental design rigorously implemented the 3Rs principle (refinement: optimized anesthesia protocols; replacement: in vitro peripheral blood mononuclear cell (PBMC) models for preliminary screening; reduction: minimal sample size calculation). Peripheral blood mononuclear cells (PBMCs) were isolated from healthy human donors using Ficoll‐Paque density gradient centrifugation (400*g*, 30 min). Cells were cultured in Dulbecco's Modified Eagle Medium (DMEM; Gibco) supplemented with 10% fetal bovine serum (FBS; Gibco), 100 U/mL penicillin, and 100 μg/mL streptomycin (Solarbio) under standard conditions (37°C, 5% CO₂).

### 2.2 In Vivo Osteomyelitis Modeling and Grouping

Animal Groups (*n* = 20/group):

**Control Group:** Under ketamine anesthesia (90 mg/kg, intraperitoneal; Jiangsu Hengrui Medicine), rats underwent sham surgery involving femoral exposure without bone injury or bacterial inoculation.

**Model Group (Osteomyelitis Model):** After anesthesia, a 2 cm lateral femoral incision was made. The femur was carefully exposed, and a standardized defect (a hole drilled with a 1.0 mm Kirschner wire) was created into the medullary cavity. A 50 μL suspension of methicillin‐sensitive *Staphylococcus aureus* (MSSA; 1 × 10⁶ CFU/mL; Nanjing Banzhen Biotech), prepared from an overnight culture grown in Tryptic Soy Broth and diluted to the target concentration in sterile saline, was injected into the medullary cavity through the created defect. The wound was then closed in layers.

**Propolis Flavonoid (PF) Group (PF‐treated Model):** Model rats (prepared as described for the Model Group) received daily oral gavage of 10 g/kg PF ( ≥ 95% purity; Sigma‐Aldrich) dissolved in saline for 6 weeks post‐infection. The treatment started 24 h after the induction of osteomyelitis.

### 2.3 In Vitro PBMC Treatment Groups and Rationale

Human PBMCs were used to investigate the direct effects of PF on T cell differentiation pathways and to explore the role of the TAZ/TEAD1 axis in a human cellular context, complementing the in vivo findings. TGF‐β1 was used to mimic an inflammatory environment that influences Treg/Th17 differentiation.

### 2.4 PBMC Groups

**Control:** PBMCs cultured with 10% healthy human serum (HHS).

**Inflammation Model:** PBMCs cultured with 15 ng/mL recombinant transforming growth factor‐β1 (TGF‐β1) (PeproTech) + 10% HHS to induce an inflammatory‐like state or skew T cell differentiation.

**Model** + **PF:** PBMCs cultured with TGF‐β1 + 10% HHS, with PF ( ≥ 95% purity; Sigma‐Aldrich) added directly to the medium to a final concentration of 10 µg/mL.

**TEAD1 + Model:** PBMCs transfected with a TEAD1 overexpression plasmid (Addgene #38175; 25 nM used for transfection optimization) prior to TGF‐β1 stimulation, to investigate TAZ‐TEAD1 interaction.

**TEAD1 + Model** + **PF:** PBMCs transfected with TEAD1 plasmid, then treated with TGF‐β1 + PF equivalent.

### 2.5 Rat PBMC Isolation and Culture

To validate the findings across species, rat PBMCs were isolated from the peripheral blood of healthy Wistar rats using Rat PBMC Isolation Kit (Solarbio) and cultured under identical conditions to human PBMCs.

## 3 Experimental Methods

### 3.1 Western Blot Analysis

Tissue samples (from bone lesions) were homogenized in RIPA lysis buffer (Beyotime) with protease inhibitors. Protein concentrations were determined via BCA assay (Pierce). Proteins (40 μg/lane) were separated on 15% SDS‐PAGE gels and transferred to PVDF membranes. Membranes were blocked with 5% non‐fat milk and incubated overnight at 4°C with primary antibodies against TAZ (1:1000, CST #70148), RORγt (1:800, Abcam #207082), FOXP3 (1:1000, CST #98377), and Glyceraldehyde 3‐phosphate dehydrogenase (GAPDH; 1:5000; Proteintech #60004‐1‐Ig) as the loading control. Chemiluminescent signals were captured using a Tanon 5200 system and analyzed with ImageJ v1.44p for relative band intensity. Protein bands were confirmed based on their predicted molecular weights. Full‐length and uncropped blot images are available in Supporting Information File 1.

### 3.2 Flow Cytometry

PBMCs (from rat peripheral blood) were stained with FITC‐anti‐CD4 (Thermo #11‐0042‐82), PE‐anti‐IL‐17 (Thermo #12‐7179‐42), and PE‐anti‐FOXP3 (Thermo #12‐4777‐42) for 30 min at 4°C. For intracellular staining of IL‐17 and FOXP3, cells were first stimulated (with PMA/Ionomycin for IL‐17) and then fixed and permeabilized according to standard protocols (using BD Cytofix/Cytoperm). After washing, cells were analyzed using a BD FACSVerse flow cytometer (BD Biosciences). Data were processed with FlowJo v10.8 (Tree Star).

### 3.3 5‐Ethynyl‐2’‐Deoxyuridine (EdU) Proliferation Assay

Periosteal cells (2 × 10³ cells/well), isolated from the femurs of rats from each experimental group, were incubated with 20 μM EdU (RiboBio) for 2 h, fixed with 4% paraformaldehyde, and permeabilized with 0.5% Triton X‐100. After Click‐iT reaction (Apollo 567 fluorescent dye; RiboBio), nuclei were counterstained with Hoechst 33342. Proliferation rates were quantified by counting EdU‐positive cells relative to total DAPI‐stained nuclei from multiple fields of view using fluorescence microscopy.

### 3.4 Enzyme‐Linked Immunosorbent Assay (ELISA) Detection

Cytokine levels (IL‐17, IL‐21, IL‐2, IL‐10) in bone tissue homogenates and TAZ, RORγt, and FOXP3 levels in PBMC lysates were measured using commercial ELISA kits (R&D Systems, Invitrogen) according to manufacturers’ protocols. Absorbance at 450 nm was read, and concentrations were calculated based on standard curves and subsequently normalized to total protein concentration of the lysate.

### 3.5 Bacterial Burden Analysis

Bone tissues were weighed, homogenized in sterile saline, and serially diluted. Aliquots were plated on tryptic soy agar and incubated at 37°C for 24 h. Colony‐forming units (CFUs) were counted and expressed as Log10 CFU/g of tissue.

### 3.6 Statistical Analysis

Data are expressed as mean ± standard deviation (SD). Normality was assessed using Shapiro‐Wilk test. One‐way ANOVA with Bonferroni post‐hoc test (SPSS v26.0, IBM) was used for intergroup comparisons. Statistical significance was set at *p* < 0.05. All experiments were performed with at least three biological replicates.

## 4 Results

To investigate the immunomodulatory effects of PF in COM, we first assessed the expression of TAZ, a key regulator in the Hippo pathway, in the bone tissue of our rat model.

### 4.1 Propolis Flavonoids Enhance TAZ Protein Expression and Reduce Bacterial Burden

**Figure 1:**
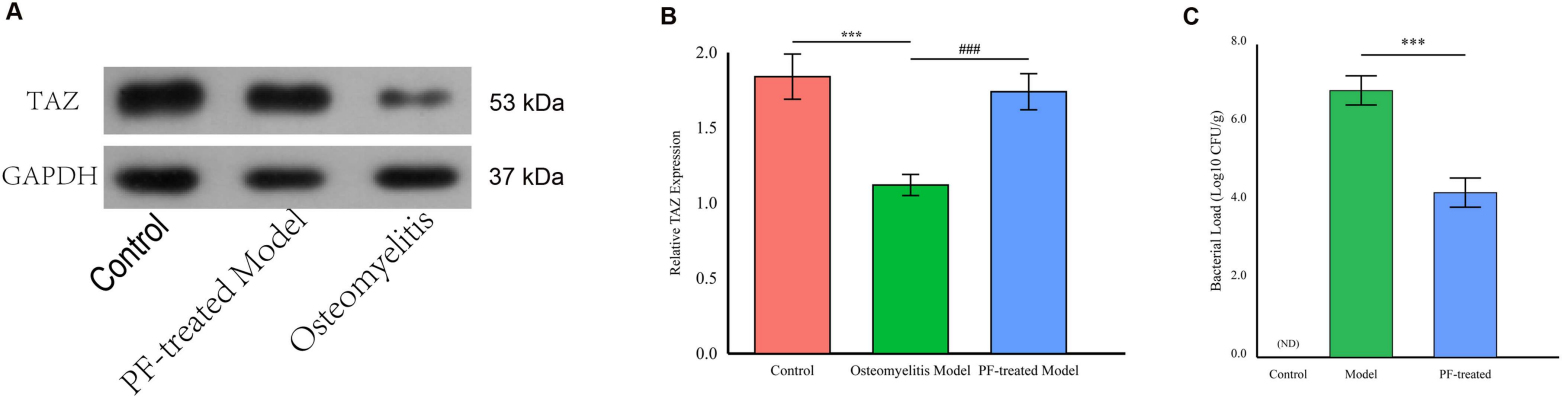
Propolis flavonoids (PF) enhance TAZ expression in osteomyelitis models. (A) Western blot analysis was performed to evaluate TAZ protein expression across groups. Representative Western blot bands of TAZ and the loading control GAPDH are shown. (B) Densitometric quantification of relative TAZ protein expression (normalized to GAPDH). (C) Bacterial load in bone tissues quantified by CFU counting (*n* = 6). Data are presented as mean ± SD, with individual data points shown. **p < 0.01, ***p < 0.001 vs. Control; ^##^ *p* < 0.01 vs. Osteomyelitis Model.

Western blot analysis revealed significant alterations in TAZ protein expression in the bone tissues across experimental groups (Figure 1A). Compared to controls (1.84 ± 0.15 relative expression), osteomyelitis models exhibited a significant reduction in TAZ protein levels (1.12 ± 0.07, *p* < 0.001). PF treatment significantly restored TAZ expression to near‐control levels (1.74 ± 0.12, *p* < 0.001 vs. model) (Figure 1B, Table 1). Additionally, microbiological analysis showed that the osteomyelitis model established a high bacterial load (6.82 ± 0.51 Log10 CFU/g). PF treatment significantly reduced the bacterial burden to 4.21 ± 0.43 Log10 CFU/g (*p* < 0.001) (Figure 1C), indicating clear antimicrobial efficacy alongside its immunomodulatory effects.

**Table 1:** TAZ Expression in Bone Tissues (*n* = 20, mean ± SD, relative expression).

Group	TAZ (relative expression)
Control	1.84 ± 0.15
Osteomyelitis model	1.12 ± 0.07
PF‐treated model	1.74 ± 0.12
F value	14.205
*p* value	< 0.001

To determine whether PF modulates Th17/Treg cell dynamics, we used flow cytometry to quantify these cell populations in peripheral blood from all groups.

### 4.2 Propolis Flavonoids Modulate Th17/Treg Cell Dynamics in Peripheral Blood

**Figure 2:**
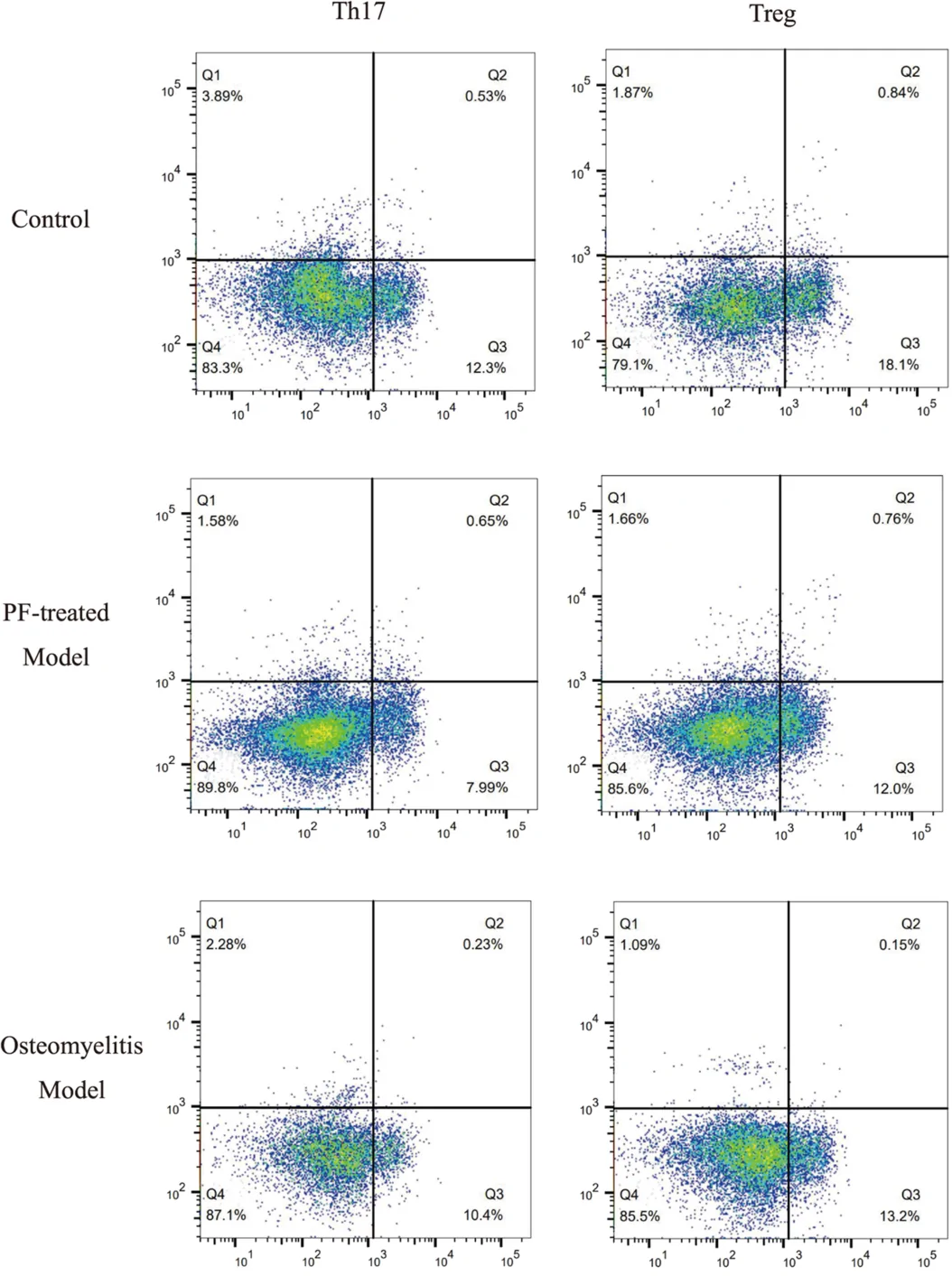
PF reverses Th17/Treg imbalance in peripheral blood of osteomyelitis models. Flow cytometry was used to analyze Th17 (CD4⁺IL‐17A⁺) and Treg (CD4⁺Foxp3⁺) cell populations in peripheral blood. Left panels depict Th17 cell distribution, while right panels show Treg cell distribution.

Flow cytometry demonstrated disease‐associated T cell polarization in rat peripheral blood (Figure 2). The model group showed suppressed Th17 populations (1.58 ± 0.31%) compared to controls (3.89 ± 0.58%, *p* < 0.001) and elevated Treg frequencies (11.5 ± 0.29%) compared to controls (1.87 ± 0.33%, *p* < 0.001). PF intervention partially reversed these trends, increasing Th17 cells to 2.28 ± 0.35% (*p* = 0.002 vs model) and reducing Tregs to 1.66 ± 0.27% (*p* < 0.001 vs model) (Table 2).

**Table 2:** Th17/Treg proportions in peripheral blood (*n* = 20, mean ± SD, %).

Group	Th17 (%)	Treg (%)
Control	3.89 ± 0.58	1.87 ± 0.33
Osteomyelitis model	1.58 ± 0.31	11.5 ± 0.29
PF‐treated model	2.28 ± 0.35	1.66 ± 0.27
F value	15.275	13.179
*p* value	< 0.001	< 0.001

Given the changes in T cell populations, we next examined the expression of their key lineage‐defining transcription factors, RORγt (Th17) and FOXP3 (Treg), in the affected bone tissue.

### 4.3 Propolis Flavonoids Affect Transcriptional Regulators RORγt and FOXP3 in Bone Tissue

**Figure 3:**
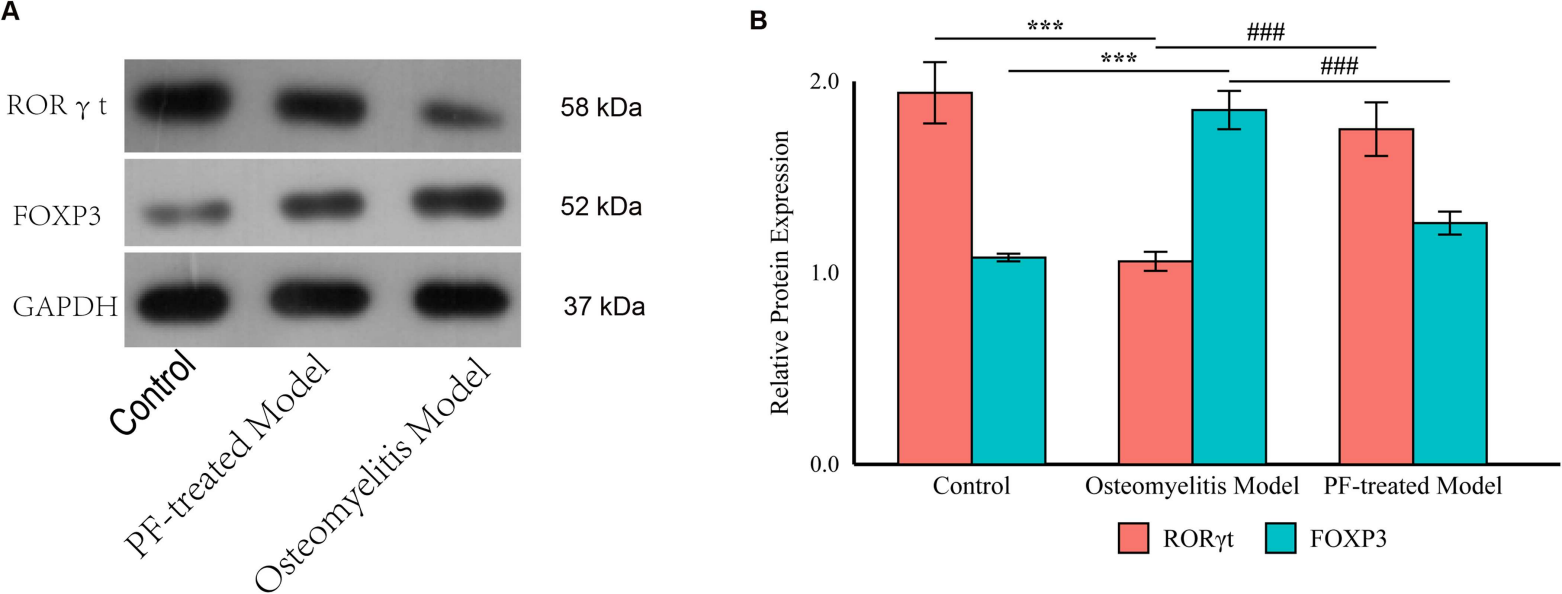
PF normalizes transcriptional regulators RORγt and FOXP3 in osteomyelitis models. (A) Western blot analysis was used to assess the expression of RORγt and FOXP3. Representative bands are displayed. (B) Densitometric quantification of relative RORγt and FOXP3 protein expression (normalized to GAPDH). Data are presented as mean ± SD, with individual data points shown. **p < 0.01 vs. Control; ^##^ *p* < 0.01 vs. Osteomyelitis Model.

The osteomyelitis model exhibited suppressed RORγt (1.06 ± 0.05) compared to controls (1.94 ± 0.16, *p* < 0.001) and elevated FOXP3 expression (1.85 ± 0.10) compared to controls (1.08 ± 0.02, *p* < 0.001) in bone tissue. PF treatment normalized these markers, increasing RORγt to 1.75 ± 0.14 (*p* < 0.001 vs model) and reducing FOXP3 to 1.26 ± 0.06 (*p* < 0.001 vs model) (Figures 3A, 3B; Table 3).

**Table 3:** RORγt and FOXP3 expression in bone tissues (*n* = 20, mean ± SD, relative expression).

Group	RORγt	FOXP3
Control	1.94 ± 0.16	1.08 ± 0.02
Osteomyelitis model	1.06 ± 0.05	1.85 ± 0.10
PF‐treated model	1.75 ± 0.14	1.26 ± 0.06
F value	13.515	11.435
*p* value	< 0.001	< 0.001

To understand the functional consequences of altered T cell balance, we measured the levels of key pro‐ and anti‐inflammatory cytokines in bone tissue lysates.

### 4.4 Propolis Flavonoids Modulate Inflammatory Cytokine Expression in Bone Tissue

**Figure 4:**
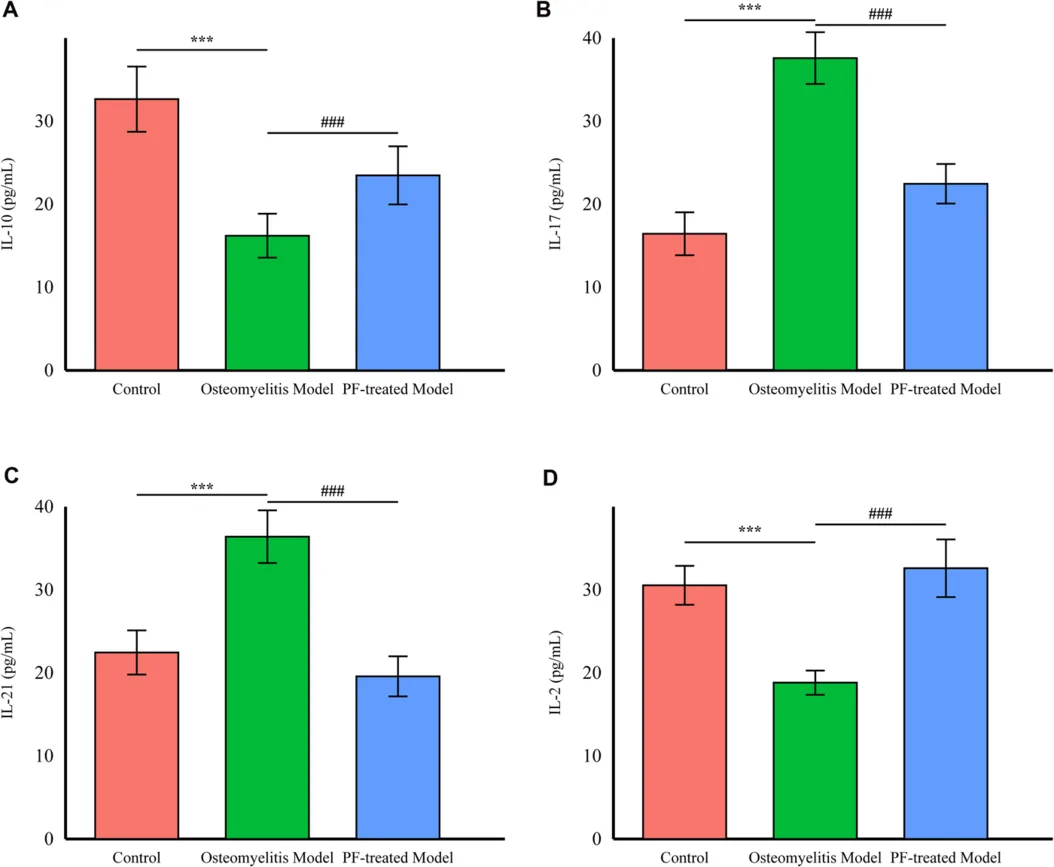
PF modulates inflammatory cytokine profiles in osteomyelitis models. ELISA was conducted to examine the expression levels of inflammatory cytokines in bone tissue homogenates. Quantification of (A) IL‐10, (B) IL‐17, (C) IL‐21, and (D) IL‐2. Data are presented as mean ± SD (pg/mL), with individual data points shown. *p < 0.05, **p < 0.01 vs. Control; ^#^ *p* < 0.05, ^##^ *p* < 0.01 vs Osteomyelitis Model.

PF treatment significantly modulated cytokine levels in bone tissue as measured by ELISA (Figure 4). The levels of pro‐inflammatory cytokines IL‐17 and IL‐21 were significantly elevated in the model group but were reduced by PF treatment (IL‐17: 22.46 ± 2.39 vs 37.59 ± 3.11 pg/mL, *p* < 0.001; IL‐21: 19.57 ± 2.41 vs 36.38 ± 3.17 pg/mL, *p *< 0.001). Conversely, the anti‐inflammatory cytokines IL‐10 and IL‐2, which were suppressed in the model group, were significantly increased by PF treatment (IL‐10: 20.54 ± 3.06 vs 14.18 ± 2.32 pg/mL, *p* = 0.001; IL‐2: 28.51 ± 3.04 vs 16.46 ± 1.28 pg/mL, *p* < 0.001) (Table 4).

**Table 4:** Cytokine profiles in bone tissues (pg/mL) (*n* = 20, mean ± SD) (Measured by ELISA).

Group	IL‐10	IL‐17	IL‐21	IL‐2
Control	28.56 ± 3.44	16.44 ± 2.58	22.44 ± 2.66	26.71 ± 2.05
Osteomyelitis model	14.18 ± 2.32	37.59 ± 3.11	36.38 ± 3.17	16.46 ± 1.28
PF‐treated model	20.54 ± 3.06	22.46 ± 2.39	19.57 ± 2.41	28.51 ± 3.04
F value	11.664	15.204	13.418	10.788
*p* value	< 0.001	< 0.001	< 0.001	< 0.001

To assess the impact of PF on bone repair capacity, we evaluated the proliferation of periosteal cells isolated from the different groups.

### 4.5 Propolis Flavonoids Promote Periosteal Regeneration

**Figure 5:**
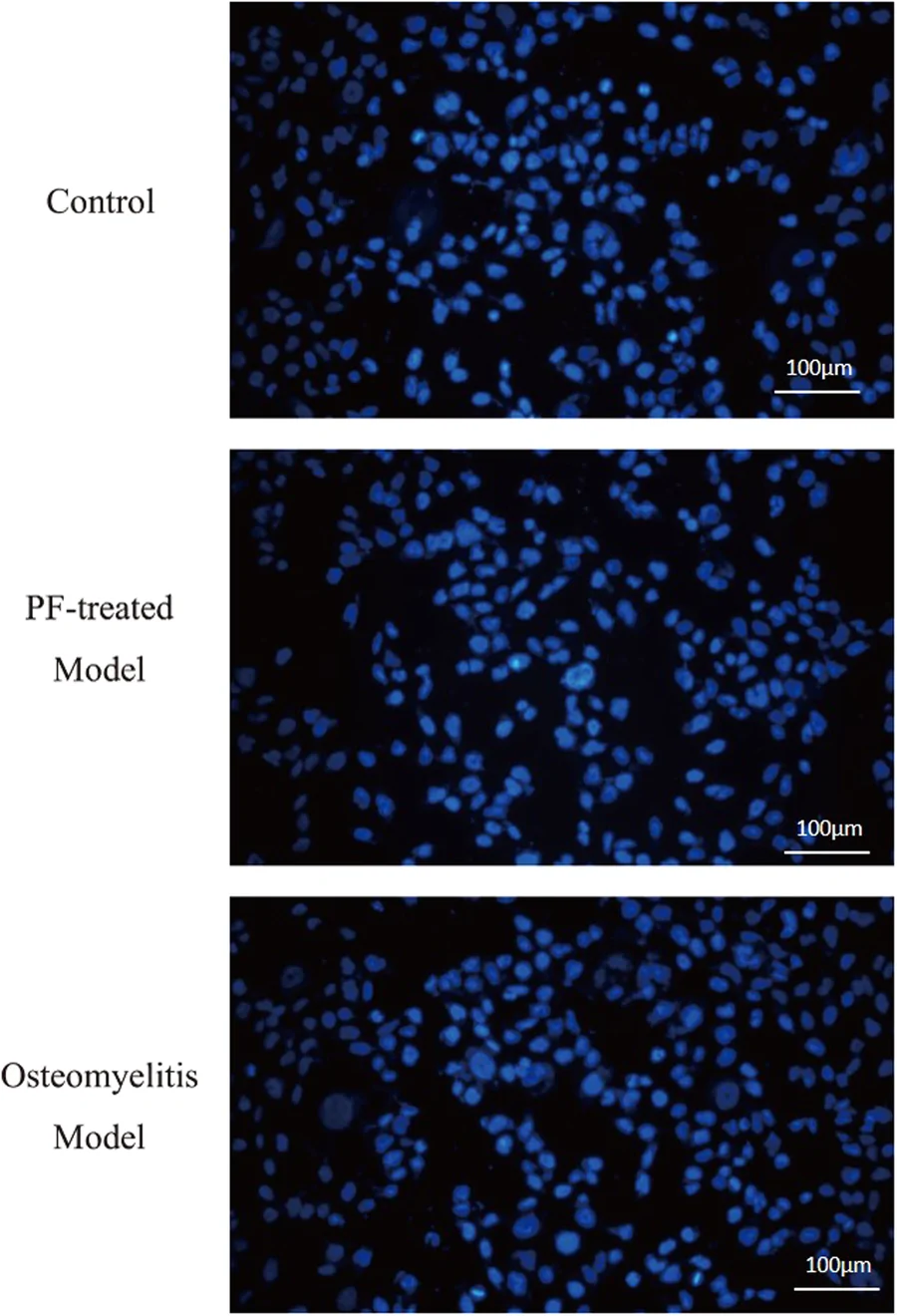
PF promotes periosteal cell proliferation in osteomyelitis models. EdU staining was employed to assess periosteal cell proliferation. Representative images are shown, with nuclei stained blue by DAPI. Magnification, 200×. Scale bar = 100 μm.

EdU assays revealed PF‐enhanced bone repair capacity (Figure 5). Periosteal cell proliferation was significantly decreased in models vs controls (*p* < 0.001), while PF treatment restored proliferation to near‐control levels (*p* = 0.002 vs. model).

Finally, to further dissect the molecular mechanism and confirm the role of the TAZ‐TEAD1 axis, we used an in vitro model with human PBMCs.

### 4.6 TAZ‐Mediated Immunoregulation in Human PBMCs

**TAZ/RORγt Dynamics:** In vitro models (human PBMCs stimulated with TGF‐β1) showed suppressed TAZ (1.03 ± 0.01 vs control 1.84 ± 0.16 pg/mL) and RORγt (1.25 ± 0.05 vs. 1.75 ± 0.14 pg/mL) as measured by ELISA. PF treatment reversed these effects (TAZ: 1.79 ± 0.15 pg/mL; RORγt: 1.83 ± 0.16 pg/mL), an effect that was significantly abolished by TEAD1 overexpression (*p* < 0.001) (Table 5).

**Table 5:** PBMC molecular markers (pg/mL) (*n* = 20, mean ± SD) (measured by ELISA).

Group	TAZ	RORγt	FOXP3
Control	1.84 ± 0.16	1.75 ± 0.14	1.13 ± 0.05
Inflammation model (TGF‐β1)	1.03 ± 0.01	1.25 ± 0.05	1.92 ± 0.17
Model + PF	1.79 ± 0.15	1.83 ± 0.16	1.25 ± 0.13
TEAD1 + Model	0.93 ± 0.01	1.05 ± 0.02	1.86 ± 0.16
TEAD1 + Model + PF	1.01 ± 0.01	1.18 ± 0.04	1.67 ± 0.14
F value	13.208	9.588	14.419
*p* value	< 0.001	< 0.001	< 0.001

**FOXP3 Regulation:** FOXP3 expression increased in TGF‐β1‐stimulated human PBMC models (1.92 ± 0.17 vs control 1.13 ± 0.05 pg/mL), which PF treatment reduced to 1.25 ± 0.13 pg/mL (*p* < 0.001). TEAD1 co‐treatment attenuated PF efficacy (1.67 ± 0.14 pg/mL vs PF alone, *p* < 0.01).

### 4.7 Validation of TAZ‐TEAD1 Mechanism in Rat PBMCs

**Figure 6:**
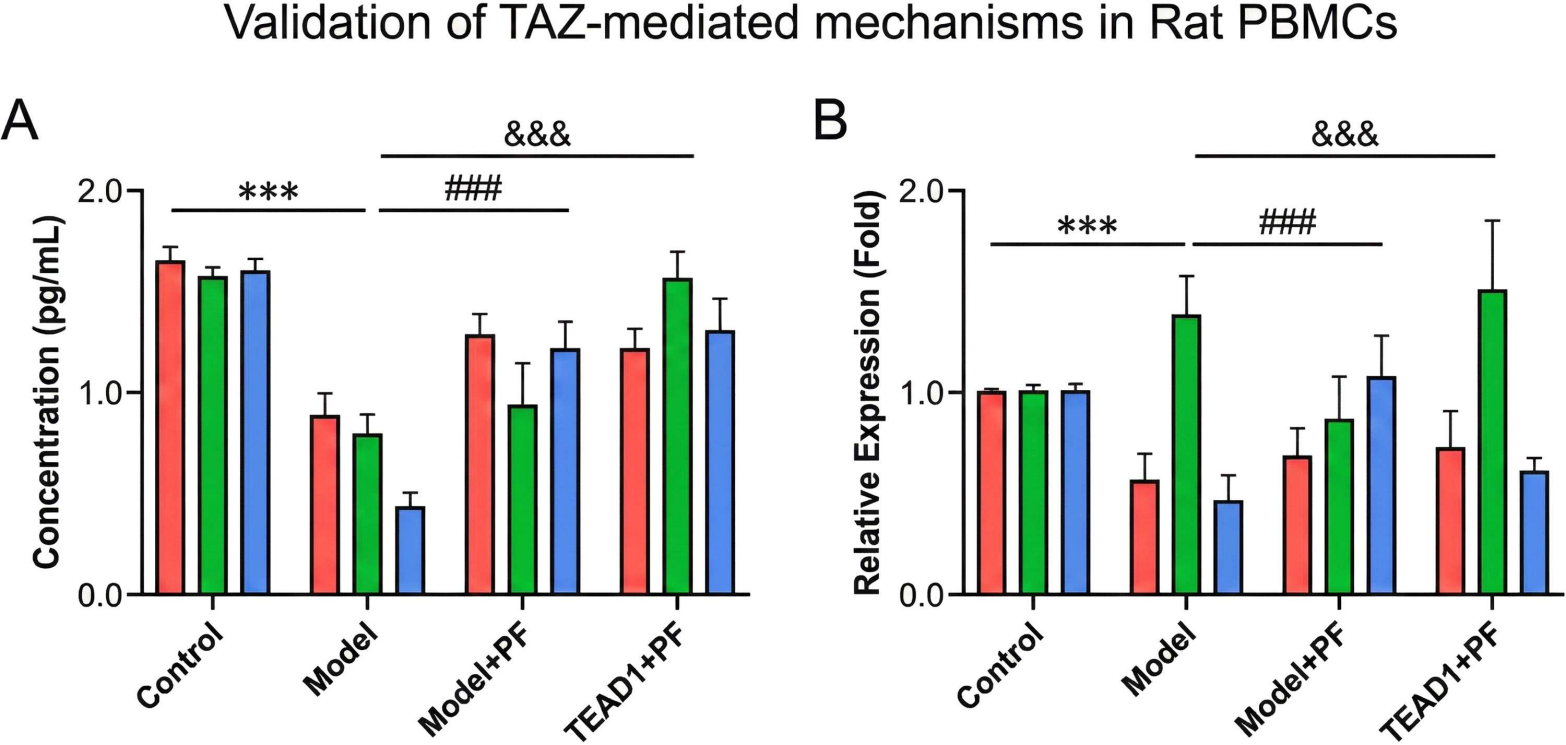
Validation of TAZ‐mediated mechanisms in Rat PBMCs. (A) Quantification of TAZ, RORγt, and FOXP3 protein levels (pg/mL) in rat PBMCs measured by ELISA. (B) Relative protein expression levels normalized to the Control group (fold change). Data are presented as mean ± SD (*n* = 6). ***p < 0.001 vs. Control; ^###^ *p* < 0.001 vs. Model; ^&&&^ *p* < 0.001 vs. Model+PF.

To bridge the biological gap between our in vivo rat model and in vitro human data, we validated the mechanism in Rat PBMCs. Consistent with human data, PF treatment significantly upregulated TAZ (1.68 ± 0.12 vs 1.05 ± 0.08 pg/mL, *p* < 0.001) and RORγt while suppressing FOXP3 in TGF‐β1‐stimulated rat PBMCs. Furthermore, TEAD1 overexpression similarly attenuated the effects of PF (Figure 6), confirming that the TAZ‐mediated immunomodulation is conserved in the rat system.

## 5 Discussion

Chronic osteomyelitis (COM) remains a formidable clinical challenge, largely driven by persistent bacterial infection and resulting immune dysregulation [26, 27]. In the pathophysiology of COM, the balance between regulatory T cells (Treg) and Th17 helper T cells plays a crucial role. This study demonstrates that propolis flavonoids ameliorate inflammation and promote cellular repair markers in a rat model of COM by modulating TAZ‐mediated Treg/Th17 homeostasis. Our key finding is that PF restores Th17 cell populations while paradoxically reducing the levels of their main effector cytokines, IL‐17 and IL‐21, suggesting a nuanced immunomodulatory role rather than simple immunosuppression.

The observed increase in Th17 cells with PF treatment, coupled with a decrease in their effector cytokines IL‐17 and IL‐21, suggests a sophisticated regulatory mechanism rather than a simple contradiction. In chronic infections like COM, a complete suppression of the Th17 response could lead to immune exhaustion and failure to control the pathogen. PF may therefore restore the Th17 cell population to a healthy level, crucial for host defense, while concurrently modulating their cytokine production to mitigate excessive inflammatory pathology. We hypothesize that this discrepancy represents a form of functional plasticity. Although we lack direct transcriptomic evidence in this study, similar uncoupling of cell numbers and effector functions has been observed in other chronic inflammatory contexts where immune responses are fine‐tuned to balance pathogen clearance and tissue protection [10, 28]. This regulatory mechanism suggests that propolis flavonoids exert their therapeutic effects by modulating TAZ to enhance Th17 cell presence, which could be beneficial in chronic infection scenarios, while simultaneously preventing runaway inflammation.

Our results show that TAZ expression is suppressed in the bone tissue of osteomyelitis models, which correlates with a suppressed Th17 phenotype (reduced Th17 cells and RORγt) and a Treg‐dominant environment. This state may represent an ineffective immune response incapable of clearing the persistent *S. aureus* infection. By upregulating TAZ, PF effectively reverses this cellular profile, driving the differentiation of Th17 cells via RORγt. This aligns with findings that TAZ signaling is a positive regulator of Th17 differentiation [24]. The PF‐induced increase in Th17 cells may restore immune vigilance against the infection, while the tempered cytokine output prevents excessive collateral tissue damage. Our data on bacterial burden (Figure 1C) confirms that this immunomodulation is accompanied by effective pathogen clearance, supporting the notion that PF restores effective immune surveillance. This finding highlights the importance of TAZ and RORγt in immune regulation and the potential of propolis flavonoids to modulate chronic inflammatory conditions through this mechanism. Such interventions with natural products targeting specific immune pathways are gaining interest [29, 30].

The in vitro experiments using human PBMCs corroborate our in vivo findings and further implicate the TAZ‐TEAD1 axis. TGF‐β1, used to mimic an inflammatory environment, suppressed TAZ and RORγt while upregulating FOXP3, a pattern that PF treatment reversed. Crucially, the overexpression of TEAD1 abolished the effects of PF. While TAZ typically acts as a co‐activator for TEADs, excess TEAD1 unbound to TAZ can function as a transcriptional repressor by recruiting co‐repressor complexes (e.g., VGL4). We propose that in our overexpression model, the surplus TEAD1 saturates DNA binding sites and, due to insufficient stoichiometric TAZ, dominantly represses RORγt transcription, thereby negating the TAZ‐mediated activation induced by PF.

However, this study has several limitations. First, while the rat model of COM is well‐established, it may not fully recapitulate the complexity of human chronic osteomyelitis. Second, propolis flavonoids are a complex mixture, and the specific flavonoid(s) responsible for the observed effects were not identified. Although a standardized extract was used, future research should focus on isolating individual components and testing their efficacy to identify the key bioactive molecules. Third, our mechanistic claim regarding the TAZ‐TEAD1 axis relies on indirect evidence from TEAD1 overexpression experiments. While these results strongly support our hypothesis, direct interventional studies using TAZ knockdown or knockout models would be required to definitively establish a causal link, and this remains a direction for future research. Fourth, our study did not include several critical endpoints for assessing therapeutic efficacy in osteomyelitis, such as radiographic analysis (e.g., micro‐CT) of bone destruction and regeneration, biomechanical testing, and detailed histopathological scoring. While our EdU assay suggests improved periosteal cell proliferation, these comprehensive assessments are essential for fully substantiating the claim of “bone repair” and represent a crucial next step for future investigations. Fifth, while TEAD1 overexpression significantly attenuated PF's effects, it did not completely abolish them. This suggests that PF likely operates through additional, TAZ‐independent mechanisms, such as the NF‐κB or MAPK pathways, which are known targets of flavonoids. Future studies should explore this “multi‐target” potential. Finally, translating these findings into clinical practice requires further investigation into optimal dosing, long‐term safety, and efficacy in human patients.

## 6 Conclusion

In conclusion, this study demonstrates that propolis flavonoids ameliorate chronic osteomyelitis by modulating the TAZ‐TEAD1 signaling axis to rebalance Treg/Th17 cell homeostasis and promote bacterial clearance. Specifically, PF restores Th17 cell populations while concurrently tempering their pro‐inflammatory cytokine production, representing a nuanced immunomodulatory mechanism. These findings identify TAZ as a key therapeutic target in COM and provide a strong mechanistic rationale for the clinical development of propolis flavonoids as a novel host‐directed therapy for this challenging disease.

## Author Contributions

**Yujie Wang:** conceptualization, methodology, writing – original draft preparation. **Chaozhu Zhang:** data curation, software. **Zeyang He:** visualization, investigation. **Liqiang Li:** writing – reviewing and editing. All authors approved the final version of the article.

## Ethics Statement

All experimental protocols were approved by the Institutional Animal Care and Use Committee of Hebei Hospital of Traditional Chinese Medicine and conducted in compliance with NIH Guide for the Care and Use of Laboratory Animals (8th ed.) and ARRIVE Guidelines 2.0. Informed consent to participate was waived because the present study does not involve any human participants.

## Conflicts of Interest

The authors declare no conflicts of interest.

## Supporting information

Supporting File

## Data Availability

Data is provided within the article files, including tables. Further enquiries can be directed to the corresponding author.
