# Supplementary material for: Propolis Flavonoids Ameliorate Chronic Osteomyelitis in a Rat Model by Modulating TAZ‐Regulated Treg/Th17 Balance
Source: Immun Inflamm Dis. 2026 Jun 4;14(6):e70432. doi: 10.1002/iid3.70432 (PMC13581067; doi:10.1002/iid3.70432)
Supplement: Supplementary file 1 [file IID3-14-e70432-s001.docx]

Original files for WB

Figure 1A

Figure 3A
